# Highlighting the success of the Canadian Journal of Pain’s inaugural Editorial Review Mentorship Program

**DOI:** 10.1080/24740527.2025.2518148

**Published:** 2025-07-10

**Authors:** M. G. Pagé, J. Katz

**Affiliations:** Department of Anesthesiology and Pain Medicine, Université de Montréal; Department of Psychology, Université de Montréal; Research Center of the Centre Hospitalier de l’Université de Montréal; Department of Psychology, York University; Department of Anesthesia and Pain Management, Toronto General Hospital; Department of Anesthesiology and Pain Medicine, University of Toronto

The *Canadian Journal of Pain* is proud to celebrate the successful completion of the first year of the Editorial Review Mentorship Program – an initiative designed to train and foster the next generation of scientific reviewers in the field of pain research. With an eye toward equity, excellence, and capacity building in scientific publishing, this program was launched to provide Program Fellows with the critical thinking and communication skills required to confidently evaluate a manuscript and to demystify the peer review process in the domain of pain research and clinical care.

In its inaugural iteration, after a call for applications, the program selected two advanced graduate students with an interest in pain research and provided them with structured mentorship from seasoned researchers and Journal Editorial Board members who had been matched with the Fellows based on common research interests and who had a reputation for submitting thorough, high-quality manuscript reviews. Over several months, Program Fellows engaged in the manuscript review process, gained hands-on experience, and received personalized feedback from their mentors. Program Fellows also met with Taylor and Francis staff as well as members of the Journal’s Editorial Office to round out their exposure to the scientific publishing industry. This initiative has proven to be a meaningful opportunity for professional development and networking.

We congratulate Ria Nishikawara and Joséanne Desrosiers, the two inaugural Fellows of the Editorial Review Mentorship Program, who showed tremendous growth during the year and produced detailed, well organized, clear and constructive manuscript reviews. Their enthusiasm, diligence, and thoughtful engagement with the peer review process exemplify the professionalism and integrity that scientific publishing depends on. We thank them for the successful completion of the Editorial Review Mentorship Program and look forward to engaging them as new reviewers for the journal! We also want to acknowledge the participation of the mentors who dedicated their time, energy and expertise to make this program a success.

We extend our sincere gratitude to Taylor and Francis, publisher of the *Canadian Journal of Pain*, for their support in this initiative. Their collaboration was central to launching and sustaining the mentorship program. We are also grateful for the support of the Canadian Pain Society that helped launch and promote this program and has always supported the development of the next generation of pain scientists and clinicians.

Looking forward, we plan to continue the Editorial Review Mentorship Program in the years ahead. Building on what we’ve learned in this first round, future iterations will include opportunities for group-based discussions, deeper exploration of equity issues,^1^ and enhancing the trustworthiness and integrity of pain research^2^ in peer review. We hope to continue to support a diverse pool of applicants and mentors, and to strengthen the peer review community to reflect the full richness of perspectives and experiences within the pain research community.

To our inaugural cohort of mentees: congratulations on your achievement! We are proud of your contributions to the *Canadian Journal of Pain* and are excited to see how your reviewing skills continue to develop and benefit the field. To our mentors and partners: thank you for your leadership, and generosity. Together, you have helped build a program that will have lasting impacts for years to come.
